# Weight Management during Pregnancy and the Postpartum Period in Women with Gestational Diabetes Mellitus: A Systematic Review and Summary of Current Evidence and Recommendations

**DOI:** 10.3390/nu15245022

**Published:** 2023-12-06

**Authors:** Jing Huang, Yi Wu, Hua Li, Hangyu Cui, Qi Zhang, Tianxue Long, Yiyun Zhang, Mingzi Li

**Affiliations:** 1 School of Nursing, Peking University, 38 Xueyuan Road, Haidian District, Beijing 100191, China; jing08234@163.com (J.H.);; 2 School of Nursing, Peking University Health Science Center, 38 Xueyuan Road, Haidian District, Beijing 100191, China

**Keywords:** gestational diabetes mellitus, weight management, systematic review, evidence summary

## Abstract

Background: Weight management during pregnancy and the postpartum period is an important strategy that can be utilized to reduce the risk of short- and long-term complications in women with gestational diabetes mellitus (GDM). We conducted a systematic review to assess and synthesize evidence and recommendations on weight management during pregnancy and the postpartum period in women with GDM to provide evidence-based clinical guidance. Methods: Nine databases and eighteen websites were searched for clinical decisions, guidelines, recommended practices, evidence summaries, expert consensus, and systematic reviews. Results: A total of 12,196 records were retrieved and fifty-five articles were included in the analysis. Sixty-nine pieces of evidence were summarized, sixty-two of which focused on pregnancy, including benefits, target population, weight management goals, principles, weight monitoring, nutrition assessment and counseling, energy intake, carbohydrate intake, protein intake, fat intake, fiber intake, vitamin and mineral intake, water intake, dietary supplements, sugar-sweetened beverages, sweeteners, alcohol, coffee, food safety, meal arrangements, dietary patterns, exercise assessment and counseling, exercise preparation, type of exercise, intensity of exercise, frequency of exercise, duration of exercise, exercise risk prevention, and pregnancy precautions, and seven focused on the postpartum period, including target population, benefits, postpartum weight management goals, postpartum weight monitoring, dietary recommendations, exercise recommendations, and postpartum precautions. Conclusions: Healthcare providers can develop comprehensive pregnancy and postpartum weight management programs for women with GDM based on the sixty-nine pieces of evidence. However, because of the paucity of evidence on postpartum weight management in women with GDM, future guidance documents should focus more on postpartum weight management in women with GDM.

## 1. Introduction

Gestational diabetes mellitus (GDM) is a common complication of pregnancy characterized by glucose intolerance with onset or first recognition during pregnancy [1]. The American Diabetes Association (ADA) defines GDM as “diabetes mellitus diagnosed at 24~28 weeks of gestation, excluding pregnant women with previously recognized diabetes mellitus or high-risk abnormalities of glucose metabolism detected early in the current pregnancy” [2]. According to the International Diabetes Federation (IDF), the prevalence of GDM in 2021 is estimated to be 16.7% worldwide [3]. With socioeconomic development, lifestyle changes, and improvements in assisted reproductive technology, the prevalence of GDM will increase as the proportion of women who are overweight or obese before pregnancy rises and the number of pregnant women of advanced maternal age increases [4,5]. GDM not only adversely affects the mother and fetus by increasing the risk of adverse pregnancy outcomes, such as pre-eclampsia, cesarean section, shoulder dystocia, preterm birth, macrosomia, and congenital malformations, but it also significantly increases a woman’s lifetime risk of several diseases, such as type 2 diabetes mellitus (T2DM), cardiovascular disease, renal disease, and cancer [6,7,8,9,10]. Therefore, it is of vital importance to manage GDM to minimize the complications.

Excessive gestational weight gain (EGWG) increases the risk of short- and long-term complications of GDM. A previous study found that pregnant women with EGWG had a higher risk of adverse pregnancy outcomes, such as large for gestational age (OR 2.06; 95%CI 1.44~2.93), macrosomia (OR 2.20; 95%CI 1.50~3.25), cesarean section (OR 1.45; 95%CI 1.13~1.87), and neonatal hypoglycemia (OR 3.80; 95%CI 1.20~12.00), compared with those with appropriate weight gain during pregnancy [11]. For each 1-unit increase in BMI during pregnancy, the risk of long-term T2DM increased by 16% (HR 1.16; 95%CI: 1.12~1.19) [12]. To make matters worse, postpartum weight retention (PWR) and obesity also pose a serious health threat to women with GDM. Women with high early PWR have more impaired metabolic profiles, less frequent breastfeeding, higher rates of depression, higher levels of anxiety, and lower quality of life in the postpartum period compared with women with early PWR < 5 kg [13]. For each 5 kg increase in postpartum body weight, the risk of long-term T2DM increased by 27% (HR 1.27; 95%CI 1.04~1.54) [12]. The above evidence suggests that weight is an important and modifiable intervention target throughout pregnancy and postpartum to reduce the risk of short- and long-term complications of GDM.

In recent years, weight management strategies for GDM including weight control, dietary modification, and appropriate exercise have received increased attention, and various medical guidance documents have been published to provide support and recommendations for healthcare providers to guide women with GDM to engage in healthy weight-related behaviors to optimize their weight during pregnancy and the postpartum period. The overall goal of these medical guidance documents is to optimize patient care based on systematic evidence [14]. However, in terms of content, there are differences in the recommendations of these literature sources, and each one does not cover precisely the same content even though the subject is the same; in terms of quality, the recommendations of more methodologically rigorous literature sources may be more scientific, and the differences in the methodological rigor may result in the dilution of high-quality recommendations; and in terms of publication time, the content of the literature may change over time, and the recommendations of more recently published research from the literature may be more appropriate for the current population [15,16]. All of the above issues may lead to uncertainty when it comes to clinical practice.

To address this issue, we conducted a systematic review of specific aspects of weight management during pregnancy and the postpartum period in women with GDM to assess, extract, and synthesize the available high-level evidence from the last five years, to provide clinical references for weight management during pregnancy and the postpartum period in women with GDM, and to facilitate more scientific decision making by healthcare providers.

## 2. Methods

In accordance with the protocol (CRD42023451857, https://www.crd.york.ac.uk), we conducted this systematic review. The report followed the Preferred Reporting Items for Systematic Reviews and Meta-Analyses (PRISMA) guidelines [17].

### 2.1. Establishment of the Problem

The initial questions were developed using the PIPOST principles, a problem development tool from the Joanna Briggs Institute (JBI) Evidence-Based Nursing Collaboration Center at Fudan University. In this study, the target population for the evidence refers to women with GDM or postpartum women with a history of GDM; the intervention measures refer to weight control (recommendations or measures for weight gain prevention, weight loss, weight management goals, weight monitoring, etc.), diet management (recommendations or measures for eating patterns, food types, energy, nutrients, etc.), and exercise management (recommendations or measures for exercise type, intensity, frequency, duration, etc.) during pregnancy and the postpartum period; professionals (evidence implementers) refer to medical staff; outcomes refer to weight (BMI changes, gestational weight gain, PWR, etc.), blood glucose (blood glucose, glycated hemoglobin, postpartum glucose metabolism outcome, long-term T2DM incidence, etc.), maternal outcomes (hypertensive disorders of pregnancy, depression, anxiety, etc.), pregnancy outcomes (mode of delivery, premature delivery, dystocia, stillbirth, premature rupture of membranes, birth injury, etc.), and neonatal outcomes (macrosomia, neonatal hypoglycemia, respiratory distress, perinatal death, etc.); settings (site of application of evidence) refer to medical institutions, postpartum rehabilitation centers, and families; and the type of study refers to clinical decisions, guidelines, recommended practices, evidence summaries, expert consensus, and systematic reviews.

### 2.2. Evidence Sources and Retrieval Strategies

Between 2018 and April 2023, we systematically searched national and international specific websites and databases, including BMJ Clinical Evidence, UpToDate, World Health Organization, Guidelines International Network, National Institute for Health and Care Excellence, Scottish Intercollegiate Guidelines Network, Agency for Healthcare Research and Quality, Queensland Health, Registered Nurses’ Association of Ontario, Canadian Medical Association: Clinical Practice Guideline, Federation International of Gynecology and Obstetrics, ADA, American College of Obstetricians and Gynecologists, Royal College of Obstetricians and Gynecologists, Society of Obstetricians and Gynecologists of Canada, Cnadian Diabetes Association, IDF, Chinese Medlive Guideline, All EBM Reviews, JBI EBP Database, MEDLINE, Embase, CINAHL, Web of Science, CNIK, Wanfang, and SinoMed. A combination of subject terms and text words was used to search in databases. The search strategy for databases and websites is described in Supplementary Table S1.

### 2.3. Inclusion Criteria and Study Selection

The inclusion criteria were as follows: (1) the subjects were women with gestational diabetes mellitus or postpartum women with a history of gestational diabetes mellitus; (2) the content or interventions relate to weight management during pregnancy and the postpartum period, including weight control (recommendations or measures for weight gain prevention, weight loss, weight management goals, weight monitoring, etc.), diet management (recommendations or measures for eating patterns, food types, energy, nutrients, etc.), and exercise management (recommendations or measures for exercise type, exercise intensity, exercise frequency, exercise duration, etc.); (3) the outcomes involved weight (BMI changes, gestational weight gain, PWR, etc.), blood glucose (blood glucose, glycated hemoglobin, postpartum glucose metabolism outcome, long-term T2DM incidence, etc.), maternal outcomes (hypertensive disorders of pregnancy, depression, anxiety, etc.), pregnancy outcome (mode of delivery, premature delivery, dystocia, stillbirth, premature rupture of membranes, birth injury, etc.), and neonatal outcomes (macrosomia, neonatal hypoglycemia, respiratory distress, perinatal death, etc.); (4) the publication types were clinical decisions, guidelines, recommended practices, expert consensus, evidence summaries, and systematic reviews; (5) published in Chinese or English; and (6) published or updated from 2018 to 2023. The exclusion criteria were as follows: (1) unable to get the full text; (2) studies without reference list; (3) duplicate publications; or (4) studies with the background restricted to COVID-19.

Based on inclusion and exclusion criteria, two researchers (H. J. and L. H.) independently screened titles and abstracts to select articles for full-text review, and then a full-text review was conducted to identify articles for inclusion in the quality assessment. Any disagreements were discussed with another researcher (W. Y.) until a consensus conclusion was reached.

### 2.4. Quality Assessment

Study quality was assessed independently by two researchers (H. J. and L. H.) according to the following instruments. Any disagreements were discussed with another researcher (W. Y.) until a consensus conclusion was reached.

#### 2.4.1. Evaluation of Guidelines

The Appraisal of Guidelines for Research and Evaluation Instrument II (AGREE II) was used to appraise the quality of the guidelines, consisting of twenty-three items and six domain [18]. The overall quality of the guidelines was graded as follows: guidelines with six domains scoring > 60% were considered graded A (high quality); guidelines with three or more domains scoring > 30% were considered graded B (average quality); and guidelines with three or more domains scoring < 30% were considered graded C (poor quality). Guidelines rated “C” were not included in this study.

#### 2.4.2. Evaluation of Systematic Reviews

Evaluation of systematic reviews was carried out using the Assessment of Multiple Systematic Reviews 2 (AMSTAR 2), which included sixteen items [19]. According to the seven key items (2, 4, 7, 9, 11, 13, and 15) selected by the AMSTAR 2 research team that affected the production of systematic reviews and the validity of their results, the quality of systematic reviews was divided into four levels: high, medium, low, and very low. Systematic reviews rated “very low” were not included in this study.

#### 2.4.3. Evaluation of Clinical Decisions, Recommended Practice, and Expert Consensus

The quality assessment instrument of the Australian JBI Evidence-Based Health Care Center for text and opinion papers was used to assess the quality of clinical decisions, expert consensus, and recommended practices [20]. The instrument consisted of six items, each rated “yes”, “no”, “unclear”, and “not applicable”. The inclusion and exclusion of articles were determined by group discussion.

#### 2.4.4. Evaluation of Evidence Summaries

Critical Appraisal for Summaries of Evidence (CASE) was used to assess the quality of evidence summaries [21]. The instrument contained ten items, each rated “yes”, “partially yes”, and “no”. The inclusion and exclusion of articles were determined by group discussion.

### 2.5. Data Extraction

Data extraction was performed by two researchers (H. J. and L. H.) independently using prespecified data extraction forms. Any disagreements were discussed with another researcher (W. Y.) until a consensus conclusion was reached. Data extracted included year of publication or update, publication country or region, authors, study type, target population, and content related to weight management during pregnancy and the postpartum period in women with GDM.

### 2.6. Data Synthesis and Classification

The qualitative synthesis and classification of evidence were also performed by two researchers (H. J. and L. H.) independently. Any disagreements were discussed in the group until a consensus conclusion was reached. Principles of synthesis: (1) if the content was consistent, evidence that was concise and easy to understand was selected; (2) if the content was complementary, it was merged based on linguistic logic; and (3) if the content was contradictory, the selection was based on the principles of prioritizing evidence-based evidence, prioritizing high-quality evidence, and prioritizing the most recently published authoritative literature. When the evidence synthesis was completed, the original study on which the relevant evidence was based was traced. According to the study design and the JBI evidence Pre-classification System (2014), the evidence level was divided into I to V levels, with I being the highest level and V being the lowest level [22].

## 3. Results

### 3.1. Search Results

**Figure 1:**
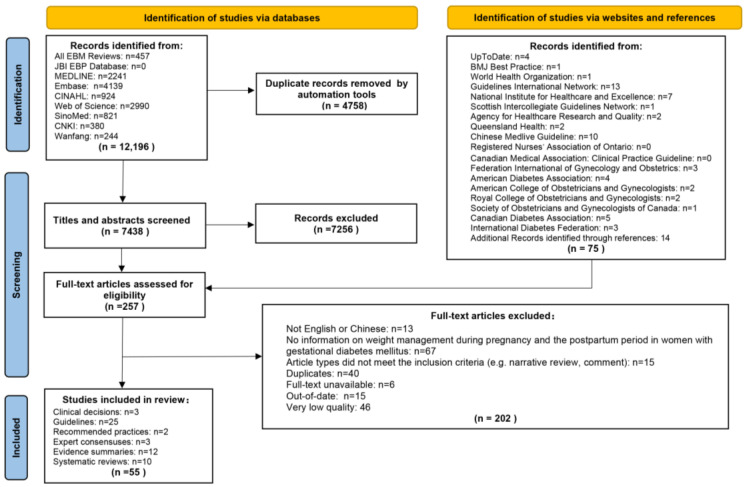
Flow diagram of literature search and screen.

A total of 12,196 articles were retrieved. After excluding 4758 duplicates, the titles and abstracts of the remaining 7438 articles were reviewed. Based on the title and abstract review, 7256 unrelated articles were excluded, leaving 182 articles. In total, 75 articles were obtained from websites and references. Therefore, a total of 257 full-text articles were reviewed. The results of the full-text review showed that 202 articles did not meet the inclusion criteria, of which 13 articles were published in languages other than English or Chinese, 67 articles did not have information on weight management during pregnancy and the postpartum period in women with GDM, 5 articles did not meet the inclusion criteria for study types, 40 articles were duplicated, 6 articles were not available for full text, 15 articles were published beyond the specified time limit, and 46 articles were rated as very low quality. Finally, we included three clinical decisions, twenty-five guidelines, two recommended practices, three expert consensuses, twelve evidence summaries, and ten systematic reviews. The flow diagram of literature search and selection is presented in Figure 1.

### 3.2. Quality Assessment

#### 3.2.1. Quality Evaluation Results of Guidelines

Of the twenty-five guidelines included in the evidence extraction, five were from the United States of America [23,24,25,26,27], three from China [28,29,30], two from the United Kingdom [31,32], two from Canada [33,34], two from Japan [35,36], two from Iran [37,38], one from Greece [39], one from India [40], one from Turkey [41], one from Pakistan [42], one from MENA region [43], one from Poland [44], one from Qatar [45], one from Australia [1], and one from Germany [46]. According to the AGREE II, the average scores of the six domains were as follows: scope and purpose = 87.78%; stakeholder involvement = 61.00%; rigor of development = 57.29%; clarity of presentation = 82.56%; applicability = 44.25%; and editorial independence = 75.50%. In this study, four guides were rated as grade A, and twenty-one were rated as grade B. The characteristics of the guidelines and the scores for each domain are shown in Supplementary Table S2.

#### 3.2.2. Quality Evaluation Results of Systematic Reviews

Of the ten systematic reviews included in the evidence extraction, there were one from the Cochrane database [47], five from the MEDLINE database [48,49,50,51,52], three from the CINAHL database [53,54,55], and one from the Embase database [56]. One study was a review of systematic reviews [47], one study included both randomized controlled trials and observational studies [53], and the original study for all other studies was randomized controlled trials [48,49,50,51,52,54,55,56]. Six systematic reviews were of women with GDM, two of which focused on lifestyle interventions [47,54], and four of which focused on dietary changes only [50,51,52,55]. Four systematic reviews were of women with prior GDM, three of which focused on lifestyle interventions [48,49,56] and one on dietary interventions only [53]. Of the ten systematic reviews, one was of high quality [47], two were of medium quality [51,56], and seven were of low quality [48,49,50,52,53,54,55]. The results of the quality assessment of the systematic reviews are presented in Supplementary Table S3.

#### 3.2.3. Quality Evaluation Results of Evidence Summaries

After a group discussion, all evidence summaries were deemed to be of acceptable quality and were included in evidence extraction, of which eight were from the JBI EBP Database [57,58,59,60,61,62,63,64], and four were from the Wanfang [65,66,67,68]. Of the twelve evidence summaries, one evidence summary focused on lifestyle interventions for GDM [62], seven focused on dietary management for GDM [58,61,63,64,65,66,68], two focused on exercise management for GDM [60,67], one focused on antenatal care for GDM [57], and one focused on education for GDM [59]. The results of the quality assessment of the evidence summaries are presented in Supplementary Table S4.

#### 3.2.4. Quality Evaluation Results of Other Studies

After group discussion, all clinical decisions, recommended practices, and expert consensus were deemed to be of acceptable quality for inclusion in the evidence extraction. A total of three clinical decisions were included, one from BMJ Best Practice [69] and two from UpToDate [70,71]. Two recommended practices and three expert consensus were included in total, two focused on GDM [72,73], one focused on the prevention of T2DM in women with prior GDM [74], one focused on exercise during pregnancy [75], and one focused on pregnancy after bariatric surgery [76]. The results of the quality assessment of the clinical decisions, recommended practice, and expert consensus are presented in Supplementary Table S5.

### 3.3. Synthesis of Evidence

We summarized sixty-two pieces of evidence about weight management of women with GDM during pregnancy, including target population, benefits, weight management goals, principles, weight monitoring, nutrition assessment and counseling, energy intake, carbohydrate intake, protein intake, fat intake, fiber intake, vitamin and mineral intake, water intake, dietary supplements, sugar-sweetened beverages, sweeteners, alcohol, coffee, food safety, meal arrangements, dietary patterns, exercise assessment and counseling, exercise preparation, type of exercise, intensity of exercise, frequency of exercise, duration of exercise, exercise risk prevention, and precautions during pregnancy. We also summarized seven pieces of evidence about postpartum weight management of women with prior GDM, including target population, benefits, postpartum weight management goals, postpartum weight monitoring, dietary recommendations, exercise recommendations, and postpartum precautions. The evidence above can be divided into four categories: weight control during pregnancy, diet management during pregnancy, exercise management during pregnancy, and comprehensive postpartum management. There were fifteen level I and fifty-four level V pieces of evidence. The evidence is summarized in Table 1. Key recommendations on weight gain goals, total energy intake, nutrients, and physical activity from the guidelines are presented in Supplementary Table S6.

### 3.4. The Proportion of Carbohydrates, Proteins, and Fats in Daily Energy Intake

The recommended proportion of nutrient intake to daily energy intake for women with GDM varied considerably across the literature. The reported percentages of carbohydrates to daily energy intake were 33~40% [72], 35~45% [39,43,73,77], 40~45 [46], 40~50% [41,44], 40~55% [27], 50% [38], and 50~60% [28]. The reported percentages of proteins to total daily energy intake were 15~20% [77], 15~25% [45], 15~30% [41], 20% [38], 20~25% [39,43], and 20~30% [44]. The reported percentages of fats to daily energy intake were 20~30% [44], 20~35% [41], 25~30% [77], 25~35% [43], 30% [38], 30~35% [46], 30~40% [27,39,45], and 40% [70]. Some literature sources recommended that saturated fat intake in women with GDM should not exceed 7% of daily energy intake [28,43,70,72], while others recommended that it should not exceed 10% of daily energy intake [27,41,44,45].

## 4. Discussion

This study reviewed and summarized the evidence and recommendations for weight management during pregnancy and the postpartum period in women with GDM. Our results found that the evidence and recommendations for weight management during pregnancy and the postpartum period for women with GDM were categorized as weight control during pregnancy, diet management during pregnancy, exercise management during pregnancy, and postpartum management. Overall, the evidence and recommendations for weight management during pregnancy in women with GDM were comprehensive, but there were differences in some areas; whereas the evidence and recommendations for postpartum weight management in women with GDM were few, and the content was general and not detailed. This systematic review provided information and references for the clinical practice of weight management during pregnancy and the postpartum period in women with GDM. The lack of postpartum weight management evidence indicated the necessity of developing high-quality guidance documents to guide the whole process of weight management of women with GDM during pregnancy and the postpartum period.

Weight management was not only the cornerstone of GDM management but also a core strategy for preventing short- and long-term complications of GDM [11,12,13]. We extracted seven pieces of evidence about weight control covering benefits, target population, principles, weight management goals, and weight monitoring. All women diagnosed with GDM should control weight according to their pre-pregnancy BMI. However, we found that the guidance documents from different countries had different recommendations for weight gain during pregnancy, which may be caused by different BMI classifications for different national populations. For example, the World Health Organization’s BMI classifications are not all the same as those commonly used in the Chinese population [28,46]. Therefore, in this systematic review, we present two different recommendations for weight gain during pregnancy for different populations. In addition, healthcare providers should record the pre-pregnancy BMI of women with GDM and assess weight changes at each of their visits, and the women themselves should monitor their weight weekly.

Diet management is one of the most crucial methods for achieving weight control goals in women with GDM. We extracted thirty-four pieces of evidence covering nutrition assessment and counseling, energy intake, carbohydrate intake, protein intake, fat intake, fiber intake, vitamin and mineral intake, water intake, dietary supplements, sugar-sweetened beverages, sweeteners, alcohol, coffee, food safety, meal arrangements, and dietary patterns. Healthcare providers should conduct a comprehensive nutritional assessment, provide nutritional counseling, and develop a personalized dietary management plan for women with GDM at their first visit. It is worth noting that women with GDM should not excessively restrict their energy intake, and their total daily calorie intake should be determined according to their pre-pregnancy BMI and stage of pregnancy. Interestingly, although only sources from the literature from the last five years were selected for this systematic review and very low-quality literature was excluded, the recommended proportion of nutrient intake to daily energy intake for women with GDM varied considerably across the literature. Dietary habits and body metabolism may vary among populations in different countries, which may explain the differences in the proportion of the above nutrients to daily energy intake in different literature studies. However, this study found differences in the ratio of nutrient intake to daily energy intake recommended in the guidance documents from the same country [28,77]. These different recommendations may lead to uncertainty in clinical implementation.

Exercise management is another crucial method for women with GDM to achieve weight control goals. We extracted twenty pieces of evidence covering exercise assessment and counseling, exercise preparation, type of exercise, intensity of exercise, frequency of exercise, duration of exercise, and exercise risk prevention. As with dietary management, professionals should conduct a comprehensive medical assessment and exercise counseling for women with GDM. However, this study found that some guidance documents listed both absolute and relative contraindications to exercise during pregnancy [34,43,67], whereas others simply listed contraindications without distinguishing between the two [1,28,77]. Pregnant women with absolute contraindications to exercise are not advised to exercise, while those with relative contraindications to exercise may exercise appropriately based on professional advice. The different delineation of contraindications to exercise by different guidelines may increase uncertainty when it comes to clinical implementation. Moderate-intensity aerobic exercise and light resistance exercise, such as walking, brisk walking, stationary biking, swimming, modified yoga, Pilates, and stretching, are appropriate for women with GDM, but exercise that may be harmful to the mother or fetus should be avoided. It is worth noting that given the different physical conditions and personal preferences of women with GDM, exercise plans should be individualized, with an emphasis on increasing physical activity and decreasing sedentary time.

Postpartum weight management in women with GDM is an effective strategy for preventing long-term complications of GDM. However, we found that there is little and generic evidence for postpartum weight control, diet management, and exercise management in women with GDM. We only extracted six pieces of evidence covering the target population, benefits, postpartum weight management goals, postpartum weight monitoring, dietary recommendations, and exercise recommendations. Laboratory and anthropometric indicators such as blood glucose, lipids, weight, and waist circumference should be tested in women with GDM at 4~12 weeks postpartum. The postpartum weight control goal for women with normal pre-pregnancy BMI is to return to pre-pregnancy weight, and the postpartum weight control goal for women with pre-pregnancy overweight or obesity is to lose 5~7% of their pre-pregnancy weight. However, we found that current guidance documents do not state the recommended time to reach postpartum weight control goals, which may lead to uncertainty in postpartum weight goal setting and diversity of postpartum weight management failure and success rates [27,38]. Regardless of whether postpartum blood glucose returns to normal or not, a healthy diet, regular exercise, and weight control should be continued, but current guidance documents do not detail how to manage diet, exercise, and control weight in the postpartum period [31].

To our knowledge, this is the first review and synthesis of evidence and recommendations related to weight management during pregnancy and the postpartum period in women with GDM. In this study, we used a robust search strategy to identify different types of guidance documents, searching not only commonly used databases but also some specific websites to maximize the selection of the entire available literature, and we selected only the literature from the last five years to ensure that the evidence and recommendations obtained were more applicable to the current population. Moreover, we used internationally recognized quality assessment tools to assess the quality of the included literature and very low-quality literature was not included in the analysis, suggesting that the sources of sixty-nine pieces of evidence were all reliable. However, we may not have retrieved all available guidance documents because we only included literature studies published in English or Chinese, which led to the omission of domestic guidance documents published in certain countries.

## 5. Conclusions

Weight management during pregnancy and the postpartum period is a crucial strategy to reduce the short- and long-term complications of GDM. The sixty-nine pieces of evidence in this study provide scientific and practical guidance for healthcare providers on weight management during pregnancy and the postpartum period for women with GDM. The paucity of evidence on postpartum weight management in women with GDM, the lack of continuity in weight management during pregnancy and the postpartum period, and the discrepancy between the recommendations of different guidance documents in the same country suggest that more comprehensive and high-quality guidance documents are needed to lead the way in the whole process of weight management in pregnancy and postpartum for women with GDM in the future.

## Figures and Tables

**Table 1:** The evidence on weight management during pregnancy and postpartum period in women with GDM.

Type of Evidence		Serial Number	Evidence	Level of Evidence
Gestational				
Weight control	Target population	1	All women diagnosed with GDM should undergo diet modification, regular exercise, and weight management [1,34,38,47,50,60,62,67,76].	I
	Benefits	2	Weight management during pregnancy in women with GDM can: (1) help achieve weight gain goals and reduce the risk of excessive gestational weight gain and postpartum weight retention; (2) increase tissue sensitivity to insulin, improve glucose tolerance, facilitate the achievement of glycemic control goals, and reduce insulin requirements; and (3) decrease the risk of adverse outcomes such as pre-eclampsia, cesarean section, large for gestational age, macrosomia, preterm labor, shoulder dystocia, and Neonatal Intensive Care Unit admission [23,24,28,33,37,46,47,50,54,67,70].	I
	Principles	3	Women with GDM should control their weight according to the recommended weight gain goals during pregnancy, and aggressive weight loss during pregnancy is not recommended [45,70].	V
	Weight control goals	4	Weight management goals during pregnancy (Institute of Medicine): (1) BMI < 18.5 before pregnancy: the total weight gain during pregnancy ranged from approximately 12.5 to 18.0 kg and the weight gain per week in the 2nd and 3rd trimester ranged from approximately 0.5 to 0.6 kg; (2) 18.5 < BMI < 24.9 before pregnancy: the total weight gain during pregnancy ranged from approximately 11.5 to 16.0 kg and the weight gain per week in the 2nd and 3rd trimester ranged from approximately 0.4 to 0.5 kg; (3) 25.0 < BMI < 29.9 before pregnancy: the total weight gain during pregnancy ranged from approximately 7.0 to 11.5 kg and the weight gain per week in the 2nd and 3rd trimester ranged from approximately 0.2 to 0.3 kg; (4) BMI ≥ 30.0 before pregnancy: the total weight gain during pregnancy ranged from approximately 5.0 to 9.0 kg and the weight gain per week in the 2nd and 3rd trimester ranged from approximately 0.2 to 0.3 kg [27,39,46,66,70].	V
		5	Weight management goals during pregnancy (Chinese Medical Association): (1) BMI < 18.5 before pregnancy: the total weight gain during pregnancy ranged from approximately 11.0 to 16.0 kg, the weight gain in the first trimester should be ≤2 kg, and the weekly weight gain in the 2nd and 3rd trimester was 0.46(0.37~0.56) kg; (2) 18.5 < BMI < 24.0 before pregnancy: the total weight gain during pregnancy ranged from approximately 8.0 to 14.0 kg, the weight gain in the first trimester should be ≤2 kg, and the weekly weight gain in the 2nd and 3rd trimester was 0.37(0.26~0.48) kg; (3) 24.0 < BMI < 28.0 before pregnancy: the total weight gain during pregnancy ranged from approximately 7.0 to 11.0 kg, the weight gain in the first trimester should be ≤2 kg, and the weekly weight gain in the 2nd and 3rd trimester was 0.30(0.22~0.37) kg; (4) BMI ≥ 28.0 before pregnancy: the total weight gain during pregnancy should be ≤9.0 kg, the weight gain in the first trimester should be ≤2 kg, and the weekly weight gain in the 2nd and 3rd trimester should be ≤0.30 kg [28].	V
	Weight monitoring	6	Professionals should record the pre-pregnancy weight, height, and BMI of women with GDM, measure their current weight at each visit and assess the change in weight from the previous visit [1,24,27].	V
		7	Women with GDM should monitor and record fasting weight early in the morning each week [46].	V
Diet management	Nutrition assessment and counseling	8	Women with GDM should receive nutritional assessment and counseling from professionals (e.g., diabetes educators, dietitians) to develop an individualized dietary management plan based on the nutritional needs of the mother and child, pre-pregnancy BMI, blood glucose levels, dietary habits, personal and socio-cultural preferences, economic levels, etc. [1,24,30,32,33,43,44,45,46,57,58,65,69,70,77].	I
		9	Nutritional assessments provided by professionals for women with GDM include but are not limited to [24,66]: (1) food, beverage, and nutrient intake, including types and amount of carbohydrate (including fiber), fat, and protein, serving sizes, and meal and snack patterns; (2) appetite and changes in appetite; (3) eating environment and meals eaten away from home; (4) diet history and behavior; (5) factors influencing access to food; (6) method of food preparation and food safety; (7) pharmacologic therapy (including insulin or oral glucose-lowering agent); (8) substance use: alcohol, tobacco, caffeine, and recreational drugs; (9) use of dietary supplements, prenatal vitamins, over-the-counter medications, complementary and/or herbal medicine; (10) knowledge, beliefs, and attitudes: motivation, readiness to change, self-efficacy, and willingness and ability to make lifestyle changes; and (11) physical activity and function: exercise patterns, functions of activities of daily living, and sleep patterns.	V
		10	The types of nutritional counseling provided by professionals for women with GDM include knowledge education, skill instruction, and individualized advice, and written educational materials should be distributed; nutritional counseling includes topics such as weight control, carbohydrate counting, Palma rule, healthy food types, healthy cooking methods, dietary records, food label reading, and physical activity [26,27,45,57,59,65,66,72,77].	I
		11	Women with GDM should receive at least three nutritional counseling sessions before delivery: (1) the 1st session (60~90 min) takes place after the diagnosis of GDM to assess and develop a dietary management plan; (2) the 2nd session (30~45 min) takes place within 1 week to assess and modify the plan; and (3) the 3rd session (15~45 min) takes place after 2~3 weeks to further assess and modification of the plan. Thereafter, counseling should be conducted every 2~3 weeks or as needed during pregnancy; and one counseling should be conducted 6~8 weeks after delivery [1,24,27,30,66].	V
		12	To assess the effectiveness of the dietary management plan, in addition to the components of the initial nutritional assessment, professionals should add the following components to the assessment at each visit for women with GDM, including but not limited to [24]: (1) daily food intake in relation to postmeal glucose readings; (2) understanding of the treatment plan for GDM; (3) weight changes compared with a previous visit; (4) self-monitoring of blood glucose records, ketone testing records (when previously recommended because of weight loss or inadequate calorie intake), and updated fetal and maternal testing or lab values.	V
		13	The form of nutritional counseling should take into account available resources and the mother’s preferences and can be performed by telemedicine if necessary, such as telephone, e-mail, and smartphone [1,59,65,66].	V
	Energy intake	14	Women with GDM should control their daily energy intake, and the total daily energy intake can be determined according to pre-pregnancy BMI. In early pregnancy, pregnant women with low weight before pregnancy need 35 kcal/kg/d, pregnant women with normal weight before pregnancy need 30 kcal/kg/d, pregnant women who are overweight before pregnancy need 25 kcal/kg/d; in the middle and third trimester of pregnancy, pregnant women with low weight before pregnancy need 40 kcal/kg/d, pregnant women with normal weight before pregnancy need 35 kcal/kg/d, and pregnant women who are overweight before pregnancy need 30 kcal/kg/d [27,28,39,41,45,69,72,73].	V
		15	Excessive restriction of energy intake (less than 1500 kcal/d) can lead to ketosis and malnutrition, which can adversely affect both the mother and the fetus; therefore, energy intake should not be less than 1600 kcal in early pregnancy, and 1800 kcal in the second and third trimesters [28,42,45,66,72].	V
		16	Women with GDM who are obese before pregnancy should reduce their energy intake by approximately 30% of the recommended intake, but ensure a minimum intake of 1600~1800 kcal/d to meet weight gain recommendations and not cause ketosis [66,70].	I
		17	The energy of breakfast, lunch, and dinner should be controlled at 10%~15%, 30%, and 30% of the total daily energy intake, respectively, and the energy of each snack can account for 5%~10% of the daily energy intake [28,65,66].	V
	Carbohydrate intake	18	Carbohydrate intake in women with GDM should not be less than 175 g per day [1,23,24,28,43,44,46,70].	I
		19	Breakfast carbohydrate intake for women with GDM should be lower than lunch and dinner. Recommended carbohydrate intake per meal: 15~30 g for breakfast, 45 g for lunch, 45 g for dinner, 15~20 g for the morning snack, 15~20 g for the afternoon snack, and 15~30 g for the bedtime snack [27,38,45,46].	V
		20	Women with GDM should give priority to complex and diverse carbohydrates, rich in dietary fiber and low GI. For example, vegetables such as lotus root, sweet potato, taro, and yam; whole grains such as brown rice, whole-wheat flour, oatmeal, buckwheat, and corn; and miscellaneous beans such as peas, red beans, and mung beans; and avoid monosaccharides such as honey [42,44,45,46,50,66,72].	V
	Protein intake	21	Daily protein intake in women with GDM should not be less than 71 or 1.1 g/kg [23,24,27,28,43,44,45,66,70].	V
		22	Women with GDM should consume equal proportions of animal and plant proteins each day. Chicken, fish, egg whites, low-fat/skimmed dairy products, legumes, and nuts are all sources of high-quality protein [43,44].	V
	Fat intake	23	Women with GDM should appropriately limit foods high in saturated fats, such as animal fats, red meats, egg yolks, coconut milk, full-fat dairy products, coconut oil, palm oil, fried foods, and butter [23,27,28,43,45,66,70].	V
		24	Unsaturated fats intake in women with GDM should account for more than 1/3 of total fat energy intake, of which 90% should be monounsaturated fats, such as olive oil, canola oil, camellia oil, and peanut oil, and 10% should be polyunsaturated fats, such as sunflower oil, corn oil, soybean oil, seeds (e.g., chia seeds, flaxseeds), nuts (e.g., walnuts), and high-fat fish (e.g., salmon, tuna, sardines) [23,27,28,43,44,45,66].	V
		25	Women with GDM should avoid foods high in trans fats such as baked goods, cakes, snacks, cookies, chips, fried foods, processed foods, and margarine [23,28,43,45,66,72].	V
	Fiber intake	26	Women with GDM should consume 25~30 g (14 g/1000 kcal/d) of dietary fiber per day. Fruits, kelp, nori, oatmeal, buckwheat noodles, konjac flour, and fresh vegetables are all foods rich in dietary fiber [23,24,27,28,38,42,43,45,66,70].	V
	Vitamin and mineral intake	27	Women with GDM should eat a varied diet, such as lean meat, chicken, fish, shrimp, low-fat/skimmed dairy products, fresh vegetables, and fruits, to ensure an adequate supply of micronutrients such as folic acid, vitamin A, vitamin B, vitamin C, vitamin D, calcium, magnesium, iron, iodine, potassium, zinc, selenium, phosphorus, and choline [1,24,28,46,66].	V
		28	Women with food deficiencies, dependence on tobacco, alcohol, or other substances, anemia, strict vegetarianism, and poor dietary habits can meet the body’s vitamin and mineral needs with dietary supplements [24,65,66].	V
	Water intake	29	It is recommended that women with GDM intake three liters of water or about ten cups per day to ensure adequate hydration; fluid intake should be increased appropriately when there is increased activity or in hot environments [27,43].	V
	Other dietary issues (dietary supplements, sugar-sweetened beverages, sweeteners, alcohol, coffee, and food safety)	30	Dietary supplements such as Omega-3 fatty acids, probiotics, and vitamin D can be used as an adjunctive management strategy for GDM [52,54,55,61,63,64,65].	I
		31	Women with GDM should reduce or avoid sugar-sweetened beverages (e.g., soft drinks and juice drinks) [66,70].	V
		32	High-intensity sweeteners can be used appropriately in women with GDM. Women are advised to choose sweeteners that are approved or generally considered safe by the US Food and Drug Administration and limit their intake to the acceptable daily intake, such as saccharin, aspartame, acesulfame potassium, sucralose, neotame, and advantame. In addition, steviol glycosides and luo han guo extracts are also generally recognized as safe when consumed within the acceptable daily intake [24,46,65,66,70,72].	V
		33	Women with GDM are advised to abstain from alcohol intake, and those who are unable to stop drinking should receive further health counseling or behavioral treatment [24,27,43,66,72].	V
		34	Caffeine should be limited in women with GDM, with a recommended daily caffeine intake of <200 mg (equivalent to 1 cup of coffee or 4 cups of black tea) [27,72].	V
		35	Women with GDM should avoid foods such as raw meat, raw eggs, and unpasteurized milk to prevent bacterial foodborne illnesses [72].	V
	Meal arrangements	36	Total daily energy, carbohydrate intake, and protein intake in women with GDM should be distributed among 3 main meals (small to medium portions) and 2~3 or more snacks (e.g., between breakfast and lunch, between lunch and dinner, and at bedtime). There should be a 2~3 h interval between meals and snacks [24,25,27,28,30,38,39,42,43,44,45,46,65,66,70,72].	V
		37	Women with GDM should eat regularly, and if a meal is missed, women are advised to test their blood glucose and choose appropriate foods to compensate [43].	V
		38	Women on insulin therapy should maintain consistency of carbohydrate intake at meals and snacks to facilitate insulin dose adjustment [70].	V
	Dietary patterns	39	Women with GDM should avoid diets that severely restrict any macronutrient, such as ketogenic diets that restrict carbohydrates, and paleolithic diets that restrict dairy products [23].	V
		40	Women with GDM should replace high-GI foods with low-GI foods. Low GI: ≤55, medium GI: 56–69, high GI: ≥70 [1,28,31,33,43,45,50,53,65,69].	I
		41	There is not enough evidence to recommend a specific dietary pattern, and professionals should provide individualized dietary plans for women with GDM [58].	I
Exercise management	Exercise assessment and counseling	42	Women diagnosed with GDM should receive a medical evaluation and exercise counseling by a professional to develop an exercise program that is tailored to the individual’s physical condition and preferences. The exercise program should include aspects such as type, frequency, duration, and intensity of exercise, and should be revised by the professional as the pregnancy progresses [1,25,37,43,60,67].	I
		43	Exercise is not recommended for women with absolute contraindications to exercise, and women with relative contraindications to exercise need to exercise moderately under the guidance of a professional [34,38,47,60,62,67,76].	V
		44	Absolute contraindications to exercise include (but are not necessarily limited to): hemodynamically significant heart conditions, restrictive lung disease, incompetent cervix/cerclage, multiple pregnancies (three or more), placenta previa after 28 weeks’ gestation, unexplained persistent vaginal bleeding, threatened preterm labor, ruptured membranes, pre-eclampsia, intrauterine growth restriction, severe anemia, uncontrolled hypertension, uncontrolled thyroid disease, and other serious cardiovascular, respiratory, or systemic disorders [1,28,34,43,67,77].	V
		45	Relative contraindications to exercise include (but are not necessarily limited to): recurrent pregnancy loss, gestational hypertension, a history of spontaneous preterm birth, mild/moderate cardiovascular or respiratory disease, symptomatic anemia, malnutrition, morbid obesity, eating disorder, twin pregnancy after the 28th week, orthopedic limitations, and other significant medical conditions [34,43,67].	V
	Exercise preparation	46	Women with GDM should exercise in a well-ventilated environment with appropriate temperature and humidity, limit exposure to ambient temperatures >32 °C, and maintain hydrotherapy pool temperatures ≤ 35 °C [28,43,67].	V
		47	Cotton socks, jogging pants, and loose clothing are recommended for exercise [28,43].	V
	Type of exercise	48	Both aerobic and resistance exercises are appropriate for women with GDM, such as walking, brisk walking, stationary bicycling, swimming (no diving), modified yoga, Pilates, stretching, jogging (for previously athletically active women), exercises using elastic bands, and pelvic floor muscle exercises. Exercise should be preceded by a warm-up and concluded with stretching exercises [27,28,30,34,39,41,42,43,44,46,67,69,72,77].	I
	Intensity of exercise	49	Moderate-intensity exercise is recommended for women with GDM [23,24,25,27,28,30,37,38,41,69].	I
		50	Women with GDM should not exercise beyond the recommended intensity. The assessment methods of exercise intensity: (1) talk test: when exercising, women can talk but not sing, suggesting that the intensity of exercise is moderate; (2) target heart rate: when exercising, the heart rate reaches 40~59% of the heart rate range (220-age), suggesting that the exercise is moderate-intensity; (3) rating of perceived exertion: when exercising at moderate intensity, the score of the rating of perceived exertion scale should not be more than 12~14 points [1,28,43,67].	V
	Frequency of exercise	51	Women without contraindications to exercise should exercise at least 5 days per week, encourage daily exercise, and avoid two or more consecutive days of inactivity [25,28,34,37,38,41,43,67,75,77].	V
	Duration of exercise	52	Women without contraindications to exercise should exercise at least 30 min per day and 150 min per week cumulatively [25,27,28,30,31,32,34,37,38,41,43,44,67,69,70,72,73].	I
		53	The 30 min of exercise per day can be divided into several 10~15 min periods [1,42,43,72].	V
		54	Exercise one hour after a meal is appropriate [31,42,46,67,72].	V
		55	Women with GDM should avoid being sedentary and spread out long periods (≥90 min) of sedentary time [77].	V
		56	For women who exercise regularly before pregnancy, it is recommended to continue to maintain appropriate exercise during pregnancy, or reduce the intensity of exercise to a moderate level; for pregnant women who do not exercise regularly before pregnancy or who are overweight/obese, it is recommended that exercise during pregnancy should start from a short period (10~15 min per day) of low-intensity exercise, and then gradually reach the recommended exercise time and intensity [1,24,28,37,38,43,44,67,77].	V
	Exercise risk prevention	57	Inappropriate exercise may lead to adverse maternal and fetal outcomes, and women with GDM should avoid: (1) positions that cause decreased venous return and hypotension, such as supine exercise (after three months of pregnancy); (2) exercises that are prone to falls, abdominal trauma, collisions, and that requires frequent changes of direction, such as boxing, basketball, soccer, horseback riding, ice skating, surfing, and cross-country bicycling; (3) exercises that can cause excessive maternal temperature, such as hot yoga and hot Pilates; (4) exercises that adds extra load to the pelvic floor, such as heavy duty strength training, bouncing, and jumping; (5) exercises at extreme altitudes, such as scuba diving, parachute jumping, mountaineering; and (6) other strenuous exercise [1,24,28,34,43,67,72].	V
		58	Exercise is not recommended for women with GDM who are hungry, unwell, or have an elevated body temperature [1].	V
		59	Women with GDM should stop exercising if they experience: high heart rate, difficulty breathing, dizziness, headache, nausea, decreased fetal movement, regular and painful contractions, vaginal bleeding, vaginal discharge, abdominal pain, back or pelvic pain, chest pain, muscle weakness that interferes with balance, or pain or swelling in the lower legs [1,28,34,43,67].	V
		60	Women who use insulin should be alert to exercise-induced hypoglycemia and avoid exercise in the early morning on an empty stomach without insulin injections. Women should carry cookies or candies with them during exercise and consume them promptly if they have symptoms of hypoglycemia [1,43,67,75].	V
		61	It is recommended that women monitor their blood sugar before and after exercise, and those with blood glucose <3.3 mmol/L or >13.9 mmol/L should stop exercising and have their urine tested for ketones [28,67].	V
Precautions during pregnancy		62	Women whose blood glucose does not reach the standard after 1~2 weeks of diet plus exercise management, or those who develop starvation ketosis after dietary adjustments, increase caloric intake, resulting in blood glucose above gestational control standards; then, insulin or medication should be added promptly [28,31,33,35,36,44,45,57,66,70,72,73,76,77].	V
**Postpartum**				
Postpartum management	Target population	63	Women with GDM should continue to practice weight management, dietary modification, and regular exercise in the postnatal period, and professionals should provide them with health education on dietary modification, physical activity, and weight control [40,43,48,49,56,74].	I
	Benefits	64	Weight management during postpartum in women with GDM can: (1) improve women’s anthropometric markers such as weight, waist circumference, and hip circumference, (2) increase insulin sensitivity and reduce women’s lifelong risk of T2DM; and (3) reduce the risk of recurrence of GDM at the time of re-pregnancy [23,25,30,31,33,45,46,48,49,56,70,71,72].	I
	Postpartum weight control goals	65	For women who were overweight or obese before pregnancy, the goal of postpartum weight management is to lose 5~7% of their pre-pregnancy weight [27,38].	V
	Postpartum weight monitoring	66	Women with GDM should undergo blood glucose screening and lipid testing at 4~12 weeks postpartum and have their blood pressure, height, weight, BMI, waist circumference, and hip circumference measured. For those with normal blood glucose results, follow-up should be conducted once every 1~3 years thereafter; for women diagnosed with pre-diabetes and diabetes mellitus, they should be treated in the endocrinology department in time [29,40,68,74,78].	V
	Dietary recommendations	67	(1) The proportion of coarse grains in staple foods should be appropriately increased, such as buckwheat, oats, and black rice; (2) cut back on sugar-rich foods, such as brown sugar, longan, red dates, and glutinous rice; (3) limit the intake of fat; 25~30 g of cooking oil per day is recommended; (4) ensure the intake of high-quality protein foods; and (5) limit the intake of fruit, about 250 g per day is recommended [29].	V
	Exercise recommendations	68	Women should get out of bed as soon as possible after delivery according to their physical condition and wound recovery, puerperal health exercises can be carried out, aerobic and resistance exercises need to be gradually increased, and it is recommended to resume strenuous exercise three months after cesarean section [29,43].	V
Postpartum precautions		69	When preparing for a second pregnancy, women with previous GDM are advised to receive preconception care and counseling from professionals to ensure adequate nutrition, healthy weight, and glucose control before, during, and after pregnancy [26].	V

## Data Availability

Data are contained within the article and supplementary materials.

## Supplementary Materials

The following supporting information can be downloaded at: https://www.mdpi.com/article/10.3390/nu15245022/s1, Table S1: Search strategy for databases and websites; Table S2: The characteristics of the guidelines and the scores for each domain; Table S3: The quality assessment of the systematic reviews; Table S4: The quality assessment of the evidence summaries; Table S5: The quality assessment of the expert consensus, recommended practices, and clinical decisions; Table S6: Key recommendations on weight gain goals, total energy intake, nutrients, and physical activity from guidelines.
