# Supplementary material for: Weight Management during Pregnancy and the Postpartum Period in Women with Gestational Diabetes Mellitus: A Systematic Review and Summary of Current Evidence and Recommendations
Source: Nutrients. 2023 Dec 6;15(24):5022. doi: 10.3390/nu15245022 (PMC10746048; doi:10.3390/nu15245022)
Supplement: Supplementary file 1 [file nutrients-15-05022-s001.zip › Table S1.pdf]

Table S1 Search strategy for databases and websites

Database/website name	Search
All EBM Reviews – Cochrane DSR, ACP Journal Club, DARE, CCTR, CMR, HTA and NHSEED	1. Diabetes, Gestational.sh.<br>2. (Diabetes, Gestational or Diabetes, Pregnancy-Induced or Diabetes, Pregnancy Induced or Pregnancy-Induced Diabetes or Gestational Diabetes or Diabetes Mellitus, Gestational or Gestational Diabetes Mellitus).ab,kw,ti.<br>3. 1 or 2<br>4. (Guideline or Guidelines or Guidance or Standard or Standards or Recommendation or Recommendations or Statement or Consensus or Systematic review or Meta-Analysis or Evidence summary).ab,kw,ti.<br>5. 3 and 4<br>6. ("2018" or "2019" or "2020" or "2021" or "2022" or "2023").yr.<br>7. 5 and 6
JBI EBP Database	1. Diabetes, Gestational.sh.<br>2. (Diabetes, Gestational or Diabetes, Pregnancy-Induced or Diabetes, Pregnancy Induced or Pregnancy-Induced Diabetes or Gestational Diabetes or Diabetes Mellitus, Gestational or Gestational Diabetes Mellitus).ab,kw,ti.<br>3. 1 or 2<br>4. (Guideline or Guidelines or Guidance or Standard or Standards or Recommendation or Recommendations or Statement or Consensus or Systematic review or Meta-Analysis or Evidence summary).ab,kw,ti.<br>5. 3 and 4<br>6. ("2018" or "2019" or "2020" or "2021" or "2022" or "2023").yr.<br>7. 5 and 6
Ovid MEDLINE	1. Diabetes, Gestational.sh.<br>2. (Diabetes, Gestational or Diabetes, Pregnancy-Induced or Diabetes, Pregnancy Induced or Pregnancy-Induced Diabetes or Gestational Diabetes or Diabetes Mellitus, Gestational or Gestational Diabetes Mellitus).ab,kw,ti.<br>3. 1 or 2<br>4. (Guideline or Guidelines or Guidance or Standard or Standards or Recommendation or Recommendations or Statement or Consensus or Systematic review or Meta-Analysis or Evidence summary).ab,kw,ti.<br>5. 3 and 4<br>6. ("2018" or "2019" or "2020" or "2021" or "2022" or "2023").yr.<br>7. 5 and 6
Embase	('pregnancy diabetes mellitus'/exp OR 'pregnancy diabetes mellitus':ab,ti OR 'diabetes mellitus gravidarum':ab,ti OR 'diabetes, gestational':ab,ti OR 'diabetes, pregnancy':ab,ti OR 'gestational

	diabetes':ab,ti OR 'gestational diabetes mellitus':ab,ti OR 'pregnancy diabetes':ab,ti OR 'pregnancy in diabetics':ab,ti) AND ('guideline':ab,ti OR 'guidelines':ab,ti OR 'guidance':ab,ti OR 'standard':ab,ti OR 'standards':ab,ti OR 'recommendation':ab,ti OR 'recommendations':ab,ti OR 'statement':ab,ti OR 'consensus':ab,ti OR 'systematic review':ab,ti OR 'meta-analysis':ab,ti OR 'evidence summary':ab,ti) AND (2018:py OR 2019:py OR 2020:py OR 2021:py OR 2022:py OR 2023:py)
CINAHL	(SU Diabetes Mellitus, Gestational OR AB Diabetes Mellitus, Gestational OR AB Diabetes, Pregnancy-Induced OR AB Diabetes, Pregnancy Induced OR AB Pregnancy-Induced Diabetes OR AB Gestational Diabetes OR AB Diabetes, Gestational OR AB Gestational Diabetes Mellitus) AND (AB Guideline OR AB Guidelines OR AB Guidance OR AB Standard OR AB Standards OR AB Recommendation OR AB Recommendations OR AB Statement OR AB Consensus OR AB Systematic review OR AB Meta-Analysis OR AB Evidence summary)
Web of Science	(TS=Diabetes, Gestational OR AB=Diabetes, Gestational OR AB=Diabetes, Pregnancy-Induced OR AB=Diabetes, Pregnancy Induced OR AB=Pregnancy-Induced Diabetes OR AB=Gestational Diabetes OR AB=Diabetes Mellitus, Gestational OR AB=Gestational Diabetes Mellitus) AND (AB=Guideline OR AB=Guidelines OR AB=Guidance OR AB=Standard OR AB=Standards OR AB=Recommendation OR AB=Recommendations OR AB=Statement OR AB=Consensus OR AB=Systematic review OR AB=Meta-Analysis OR AB=Evidence summary)<br>Limit: 2018-2023
SinoMed	1. "Diabetes, Gestational"[Unweighted:Expanded]<br>2. "Diabetes, Gestational"[Common Field:Intelligent] OR "Pregnancy-Induced Diabetes"[Common Field:Intelligent] OR "Gestational Diabetes"[Common Field: Intelligent]<br>3. (#2) OR (#1)<br>4. "Guideline"[Common Field:Intelligent] OR " Standard"[Common Field:Intelligent] OR "Recommendation"[Common Field:Intelligent] OR "Statement"[Common Field: Intelligent] OR "Consensus"[Common Field:Intelligent] OR "Systematic Review"[Common Field:Intelligent] OR "Meta-Analysis"[Common Field:Intelligent] OR "Evidence Summary"[Common Field:Intelligent]<br>5. (#3) AND (#4)<br>6. ((#3) AND (#4)) AND 2018-2023 [Date]
CNKI	(SU%=Diabetes, Gestational OR TKA=Diabetes, Gestational OR TKA=Pregnancy-Induced Diabetes OR TKA=Gestational Diabetes)

	AND (TKA=Guideline OR TKA=Standard OR TKA=Recommendation OR TKA=Statement OR TKA=Consensus OR TKA=Systematic Review OR TKA= Meta-Analysis OR TKA=Evidence summary)<br>Limit: 2018-2023
Wanfang	((Subject: (Diabetes, Gestational) or Title/Keyword: (Diabetes, Gestational or Pregnancy-Induced Diabetes or Gestational Diabetes)) and (Title/Keyword: (Guideline or Standard or Recommendation or Statement or Consensus or Systematic Review or Meta-Analysis or Evidence Summary)) and Date:2018-*
Up to Date	• Gestational Diabetes<br>• Diabetes in Pregnancy
BMJ Best Practice	• Gestational Diabetes<br>• Diabetes in Pregnancy
World Health Organization (WHO)	https://www.who.int/publications/who-guidelines<br>• Gestational Diabetes<br>• Diabetes in Pregnancy
Guidelines International Network (GIN)	https://g-i-n.net/international-guidelines-library<br>• Gestational Diabetes<br>• Diabetes in Pregnancy
National Institute for Healthcare and Excellence (NICE)	https://www.nice.org.uk/guidance<br>• Gestational Diabetes<br>• Diabetes in Pregnancy
Scottish Intercollegiate Guidelines Network (SIGN)	https://www.sign.ac.uk/search-results<br>• Gestational Diabetes<br>• Diabetes in Pregnancy
Agency for Healthcare Research and Quality (AHRQ)	https://www.ahrq.gov/gam/index.html<br>• Gestational Diabetes<br>• Diabetes in Pregnancy
Queensland Health (QLD)	https://www.health.qld.gov.au/clinical-practice<br>• Gestational Diabetes<br>• Diabetes in Pregnancy
Chinese Medlive Guideline	https://guide.medlive.cn/<br>• Gestational Diabetes<br>• Diabetes in Pregnancy
Registered Nurses' Association of Ontario (RNAO)	https://rnao.ca/<br>• Gestational Diabetes<br>• Diabetes in Pregnancy

Canadian Medical Association: Clinical Practice Guideline	https://joulecma.ca/cpg/homepage<br>• Gestational Diabetes<br>• Diabetes in Pregnancy
Federation International of Gynecology and Obstetrics (FIGO)	https://www.figo.org/<br>• Gestational Diabetes<br>• Diabetes in Pregnancy
American Diabetes Association (ADA)	https://diabetesjournals.org/<br>• Gestational Diabetes<br>• Diabetes in Pregnancy
American College of Obstetricians and Gynecologists (ACOG)	https://www.acog.org/<br>• Gestational Diabetes<br>• Diabetes in Pregnancy
Royal College of Obstetricians and Gynecologists (RCOG)	https://www.rcog.org.uk/<br>• Gestational Diabetes<br>• Diabetes in Pregnancy
Society of Obstetricians and Gynecologists of Canada (SOGC)	https://www.sogc.org/<br>• Gestational Diabetes<br>• Diabetes in Pregnancy
Canadian Diabetes Association (CDA)	https://www.diabetes.ca/<br>• Gestational Diabetes<br>• Diabetes in Pregnancy
International Diabetes Federation (IDF)	https://www.idf.org/<br>• Gestational Diabetes<br>• Diabetes in Pregnancy
